# Supplementary material for: Prehistoric population expansion in Central Asia promoted by the Altai Holocene Climatic Optimum
Source: Nat Commun. 2023 May 29;14:3102. doi: 10.1038/s41467-023-38828-4 (PMC10227073; doi:10.1038/s41467-023-38828-4)
Supplement: Supplementary file 1 — Supplementary Information [file 41467_2023_38828_MOESM1_ESM.pdf]

Supplementary Information for:

Prehistoric population expansion in Central Asia promoted by the Altai Holocene Climatic Optimum

Lixiong Xiang[1], Xiaozhong Huang[1*], Mingjie Sun[1,2], Virginia N. Panizzo[2*], Chong Huang[1], Min Zheng[1], Xuemei Chen[3], Fahu Chen[4,5]

1. Key Laboratory of Western China's Environmental Systems (Ministry of Education), College of Earth and Environmental Sciences, Lanzhou University, Lanzhou, 730000, China
2. Centre for Environmental Geochemistry, School of Geography, University of Nottingham, Nottingham, NG7 2RD, UK
3. Northwest Institute of Eco-Environmental and Resources, Chinese Academy of Sciences, Lanzhou, 730000, China
4. Alpine Paleoecology and Human Adaptation Group (ALPHA), Institute of Tibetan Plateau Research, Chinese Academy of Sciences, Beijing 100101, China
5. State Key Laboratory of Tibetan Plateau Earth System, Resources and Environment (TPESRE), Beijing 100101, China

*Corresponding Author:

Xiaozhong Huang, Email: xzhuang@lzu.edu.cn

Virginia N. Panizzo, Email: Virginia.Panizzo@nottingham.ac.uk

Supplementary Information Notes

Supplementary Note 1: Study area settings

Kanas Lake (48°11′–49°11′N, 86°23′–88°05′E; 1370 m above mean sea level (m a.s.l.)) is a freshwater alpine lake located within the southern Altai Mountains in the northernmost part of Xinjiang, China (Fig. 1a, b). Data from Habahe County meteorological station, collected during 1980–2010 CE, show that the mean annual air temperature (MAT) in the lake area is 5.3 °C, and the mean annual precipitation (MAP) is ~205.3 mm (http://www.data.cma.cn/). However, the MAP in the Kanas Lake area is ~400–700 mm, with a relatively high proportion being winter snowfall. Kanas Lake is ~24 km long with the maximum width of 2.6 km, surface area of 45.73 km^2, maximum depth of 197 m, and mean depth > 100 m[1] (Fig. 1). The lake water has a pH of 8.17 and salinity of 0.04 g/L[1]. The only inflowing river, the Kanas River, is sourced in Khüiten Peak (also called Youyi Peak in Chinese: 4374 m a.s.l.), and it then flows southwestward to the Burqin River, a major secondary tributary of the Irtysh River. The lake basin was formed by glacial erosion and dammed by a terminal moraine during the Last Glacial; the terminus of the Kanas glacier in 2009 CE was at ~2460 m a.s.l.[2]. The dominant diatom species at the present-day are the planktonic *Pantocsekiella gordonensis* and the benthic *Achnanthidium minutissimum*.

Tiewaike Lake (48°49′N, 87°00′E) is situated on the western slopes of Kanas Lake, at the altitude of 1524 m a.s.l. (Fig. 1c). Although it is a small, closed-basin alpine lake with no stream inputs or outputs and a surface area of approximately 0.02 km^2, Tiewaike Lake is considered a freshwater lake due to its low levels of chemical weathering in the catchment and the relatively low input of soluble chemical substances. The lake has a

weakly alkaline pH of 8-9 and a salinity of 0.08 g/L[3], with a maximum depth of 5.6 m. The lake is surrounded by an extensive wetland, with the surrounding trees including *Abies sibirica*, *Picea obovata*, *Pinus sibirica*, *Larix sibirica, Betula pendula,* and *B. pubescens*.

Supplementary Note 2: Sedimentary chronology

The age model for Kanas Lake is based on accelerator mass spectrometry (AMS) ^{14}C dating of 7 samples of terrestrial plant remains (TPR) selected from the top 156 cm of core KNS15D (Supplementary Tab. 1). The dated materials from Kanas Lake are terrestrial plant remains (e.g., tree twigs, bark, and stem material). A previous study showed no reservoir effect for the TPR[4], and the chronology indicates that core KNS15D spans the last ~15 kyr (Supplementary Fig. 1a). The temporal sampling resolution for core KNS15D is 25-278 yr/cm (average ~98 yr/cm). For Tiewaike Lake, the 21 AMS radiocarbon dates are listed in Supplementary Tab. 2. Among these samples, 7 dates are from aquatic plant remains (APR) and the remaining 14 are from samples of bulk organic matter (BOM). Li et al.[3] compared the ^{210}Pb and AMS ^{14}C ages of APR from the core depth of 4.5 cm in Tiewaike Lake. The age difference was 709 yr, which was ascribed to the reservoir effect for APR; thus, we used the age of 709 yr as the reservoir effect for the APR in core TWK15A. The radiocarbon reservoir effect for the BOM was estimated by comparing the ^{14}C ages of APR from the depths of 250 cm, 374 cm, and 479 cm with the ^{14}C ages of BOM from the same depths (Supplementary Tab. 2), which indicated a highly significant linear relationship (r^2=0.997) (Supplementary Fig. 14). Therefore, we calculated the ages of all the BOM samples after removing different carbon reservoir effects and then established the age-depth model for Tiewaike Lake (Supplementary Fig. 1b). The reservoir effect for

the BOM samples from Tiewaike Lake shows a long-term trend of increase through the Holocene, indicating that older carbon entered Tiewaike Lake from the catchment during the middle to late Holocene, likely due to the influence of the continuously wetting climate in the southern Altai region and the resulting enhanced soil erosion[5]. The temporal sampling resolution for core TWK15A is ~4–71 yr/cm (average ~17 yr/cm).

Supplementary Note 3: Environmental interpretation of $\delta^{30}Si_{diatom}$ at Kanas Lake

During biomineralization, diatoms discriminate against the heavier ^{30}Si isotope in favor of the lighter ^{28}Si isotope, resulting in the enrichment of the residual dissolved silicon (DSi) pool in the heaver ^{30}Si isotope. Continued diatom growth from this increasingly isotopically-heavy dissolved silicon pool causes an increase in $\delta^{30}Si_{diatom}$. Therefore, changes in $\delta^{30}Si_{diatom}$ can reflect the surface water DSi utilization in lakes[6], assuming the lake is a closed system, which often applies to lakes with periods of strong stratification. It has been demonstrated that ^{28}Si is preferentially released during diatom dissolution (with a fractionation effect of -0.55 ± 0.05‰), potentially altering the measured values of $\delta^{30}Si_{diatom}$ when the dissolution varies by >20% between individual samples[7]. No diatom dissolution is inferred for Kanas Lake due to the excellent preservation of diatoms in both the isotope samples and sedimentary diatom assemblages[8-9], suggesting that inter-sample differences in dissolution were below the 20% threshold over the analyzed interval.

The $\delta^{30}Si_{diatom}$ composition of sediments is often affected by multiple factors in lake systems, including changes in DSi concentrations and/or compositions caused by chemical weathering in the catchment (including clay dissolution and neo-formation of Si-minerals[10], river/aeolian inputs, lake water residence time, changes in stratification/overturning, and

other physical characteristics[11-15]). These factors are usually associated with changes in climate, and it has been demonstrated that in closed lake systems (e.g., Huguangyan Marr Lake), $\delta^{30}Si_{diatom}$ is a palaeotemperature proxy[16]. In the context of this study, we interpret the $\delta^{30}Si_{diatom}$ signatures as indicating changes in catchment weathering and/or periods of lake overturn (intensity of stratification), and thus climate-driven processes within the Kanas Lake catchment.

The sediments from Tiewaike Lake are rich in organic matter, and some clay-rich intervals also occur. The lake water is relatively rich in humic acids derived from the peaty forest soils in the catchment. BSi was relatively low prior to 3.6 kyr (<5%) (Supplementary Fig. 16), when the sedimentary organic matter content was high. BSi may also be influenced by the humic acids within the lake water, in addition to climate change. The sediments of Tiewaike Lake have a low diatom concentration and there is a high proportion of Chrysophytes. This would make the potential to isolate the two forms of biogenic silica very difficult, via heavy density separation and/or sieving. Not being able to do this would effectively would adversely affect the $\delta^{30}Si_{diatom}$ record. Our measurements of BSi indicated that the lowest values, together with the almost complete absence of diatoms, occurred before ~6.5 kyr. During ~6.5–3.6 kyr, the BSi values were moderately low and fluctuated between ~2.5 and ~4.0%. After 3.6 kyr, BSi increased (which is the opposite trend to that at Kanas Lake), suggesting that while the neoglaciation in the Altai Mountains had a major influence on Kanas Lake, it had little impact on the small, closed Tiewaike Lake basin.

Supplementary Note 4: XRF results and interpretation

We present the core-scanning X-ray fluorescence (XRF-scanning) results for cores KNS15D and TWK15A. For Kanas Lake, conventional XRF measurements were also conducted (Supplementary Fig. 3), and for 8 elements there was a significant positive relationship ($p < 0.01$) between the XRF-scanning and conventional XRF results. However, the relationship for Si is negative, because conventional XRF measurements can detect biogenic silica, but XRF-scanning cannot (Supplementary Fig. 4); therefore, SiO_2 in this study was measured by conventional XRF analysis. The results of a PCA of the XRF-scanning data showed that two element groups could be defined, expressed by the first two principal components (PC1 and PC2) which respectively explain 45.4% and 32.7% of the total variance in element compositions (Supplementary Fig. 5). Group I comprises both lithogenic elements (Al, K, Ti) and Zn, Cl and S, and the stratigraphic variations of these elements are expressed by sample scores on PC1. Ca also co-varies with the lithogenic elements (Supplementary Fig. 5). The sample scores on PC1 show a decreasing trend during ~11–6 kyr, followed by a slight increase. Group II mainly comprises elements associated with the detrital terrigenous fraction, including Fe, Sr, Zr, Rb, and Mn, the stratigraphic variations of which are expressed by the sample scores on PC2 (Supplementary Fig. 3 and Supplementary Fig. 5). In contrast, Zr and Sr vary inversely with Mn, Rb and Fe, with relatively low concentrations of these elements during the last deglaciation (15.0–11.7 kyr), highest concentrations in the early Holocene, and slightly decreasing concentrations in the middle to late Holocene (Supplementary Fig. 3 and Supplementary Fig. 5).

Al, Ti, Rb and K are lithogenic elements associated with the detrital mineral fraction, whereas Ca, Si and Sr represent authigenic lake production and/or allogenic inputs[17-18]. However, Ca could be of detrital origin because it covaries with Al and Ti in the sediments of Kanas Lake. In some contexts, Si can be detrital, and BSi is derived from phytoliths, or even abiotic silica produced by the weathering of silicate minerals like quartz[19]. In Kanas Lake, SiO_2 shows the opposite trend to that of the allogenic elements (Supplementary Fig. 3), and the SiO_2/Al_3O_2 ratio and SiO_2 show similar trends (r^2=0.94; Supplementary Fig. 6); SiO_2/Al_3O_2 and SiO_2 are strongly correlated with BSi (r^2=0.75 and r^2=0.65, respectively). The silica in lakes is often of biogenic origin[19], with BSi derived from diatoms, chrysophytes, radiolaria, and siliceous sponges. During diatom analysis, a small number of siliceous sponges were occasionally observed, but we assume that the Si record mainly reflects the productivity of the dominant diatom community.

In Kanas Lake, the Rb/Sr ratios are highly correlated with Rb (r^2=0.68) and weakly correlated with Sr (r^2=0.26) (Supplementary Fig. 7). The Rb/Sr ratios are therefore predominantly controlled by Rb activity during weathering within the lake watershed, indicating that the basin is mainly affected by physical erosion rather than chemical weathering[20-21]. Physical weathering increases during cold periods when the vegetation cover is reduced and greater amounts of unweathered terrestrial detrital are transported to the lake[22]. During 11.7–10.6 kyr (Geochemical Zone 2-1), Rb/Sr decreased and the sample scores on PC2 of the XRF core-scanning data increased rapidly (Supplementary Fig. 3), indicating the increased input of coarse-grained terrigenous material, which we infer was supplied by surface runoff, most likely due to the melting of local high-altitude glaciers and permafrost with the onset of the regional climatic amelioration during the early

Holocene. The trends in Sr and Zr closely track the changes in grain size (Supplementary Fig. 3), indicating that physical erosion in the catchment was the major source of these elements in the lake basin. This is supported by the evidence of clay-rich layers (indicative of higher concentrations of Fe and Rb) which are negatively correlated with intervals of higher Sr and Zr. Such erosional products (indicated by high concentrations of Sr and Zr) have been shown to be concentrated within the sand fraction[17].

In the lake environment, hypolimnetic anoxia can alter the cycling of redox-sensitive elements. Although both Fe and Mn are soluble under reducing conditions, Mn is usually more soluble than Fe, and thus the Mn/Fe ratio can be used as a palaeo-redox proxy[23 24]. Higher Mn/Fe ratios mainly reflect weaker stratification[23], as a result of, for example, lower water level and/or higher wind speed[17, 25-26]. In Tiewaike Lake, the increased Mn/Fe ratios (Supplementary Fig. 10) may reflect the predominantly oxidizing lake status at depth during 8.2–6.5 kyr, together with a lower water level, ignoring the possible effect of changes in wind speed. A shift to lower Mn/Fe ratios may therefore point to a lowering of the oxygen content of the bottom water during enhanced stratification due to high temperatures, and/or to deoxygenation caused by the decomposition of organic matter following the enhanced biological productivity during 6.5–3.6 kyr.

Supplementary Note 5: Sedimentary organic matter and its environmental significance

The $\delta^{13}C_{org}$ and C/N values of modern plants obtained in previous research have revealed significant differences between aquatic and terrestrial plants[27]. High $\delta^{13}C_{org}$ values in aquatic plants are common in lakes with longer residence times, enabling aqueous C to

equilibrate with ^{13}C-enriched atmospheric CO_2[19]. The terrestrial vegetation in the study area is dominated by C_3 plants which have more negative $\delta^{13}C_{org}$, ranging from -37 to -24‰, with the average of -27‰[3, 28-29]; while for submerged plants, $\delta^{13}C_{org}$ values vary between -20 and -12‰[30]. Moreover, other evidence shows that the lower (more negative) the $\delta^{13}C$ value, the greater the summer rainfall, and vice versa[31]. In Tiewaike Lake, $\delta^{13}C_{org}$ indicates a mixture of aquatic and terrestrial plants. In Tiewaike Lake, an increased proportion of terrigenous organic matter would result in more negative $\delta^{13}C_{org}$, while an increase in the proportion of aquatic plants would result in more positive $^{13}C_{org}$. Accordingly, the co-occurrence of higher $\delta^{13}C_{org}$ and lower C/N ratios (e.g., during ~8.2–6.5 kyr) would indicate a more significant aquatic carbon source and/or a longer lake water residence time (Fig. 2g, Supplementary Fig. 10 and Supplementary Fig. 13b). Additionally, the analysis of sediment lithology and organic matter content suggests that the abundance of aquatic plant and organic matter from its upland forest has resulted in the formation of peat sediments over time (Supplementary Fig. 13b). The pollen data of Kanas Lake and peat investigation suggested that the forest vegetation has developed and peat has been accumulating since around 8 kyr (e.g.,[4-5, 32]). Oligotrophic alpine/Arctic lakes are often N-limited, and most of their nitrogen influx is from atmospheric sources such as precipitation and snow melt[33-34]. The $\delta^{15}N_{org}$ values in the Tiewaike Lake sequence range between -2.2‰ and 0.94‰ (Supplementary Fig. 10), which further suggests that atmospheric nitrogen ($\delta^{15}N$ ~0‰) is the dominant N source[35]. During ~8.2–6.5 kyr, the lowest $\delta^{15}N_{org}$ values (from -0.6‰ to -2.2‰, average -1.4‰; Supplementary Fig. 10) and highest $\delta^{13}C_{org}$ are likely to reflect a dominant aquatic source. The shallow water level and reduced detrital inputs may result in the benthos making a much larger contribution to the total lake primary

production[33]. During 6.5–3.6 kyr, the high $\delta^{15}N_{org}$ values may have resulted from increased detrital inputs (e.g., Ti, Rb, and Rb/Sr; Supplementary Fig. 10, Supplementary Fig.11 and Supplementary Fig. 12), driven by higher terrigenous productivity (the range of $\delta^{15}N$ for terrestrial plants is 4.5–10.3‰[35-36]).

Supplementary Information Figure and Table

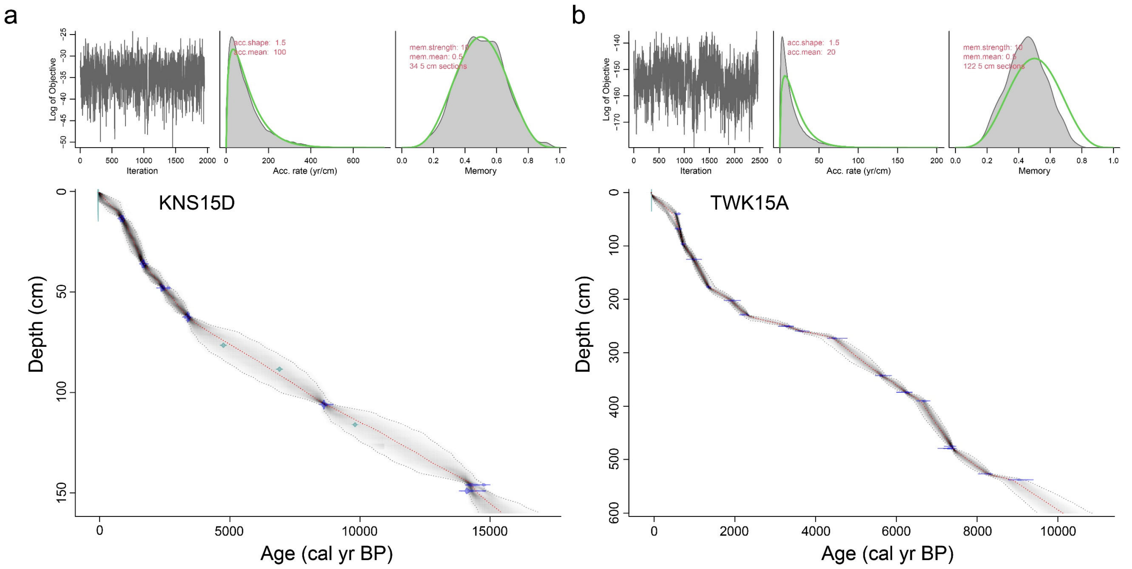

Supplementary Figure 1. Age-depth models for (a) core KNS15D from Kanas Lake and (b) core TWK15A from Tiewaike Lake, obtained using Bacon.R software. The blue dots indicate calibrated ^{14}C dates included in the age-depth model, while the green dots indicate dates that were excluded from the model. The red dashed curve shows the mean model, and the gray shaded bands are 95% confidence intervals. The radiocarbon dates are presented in Supplementary Tables 1 and 2.

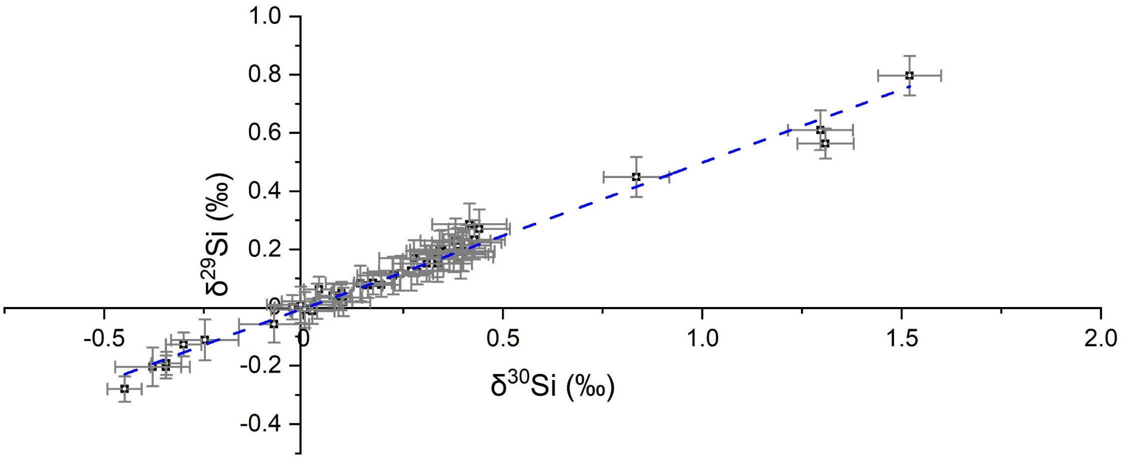

Supplementary Figure 2. Scatter plot of δ^{29}Si versus δ^{30}Si for all the silicon isotope data (n=46) obtained in this study. The data fall within the 2-sigma absolute analytical uncertainty of the mass dependent fractionation line (dashed; δ^{29}Si =0.502δ^{30}Si), which is good agreement with the kinetic fractionation factor of 0.5092 for Si[37]. Source data are provided as a Source Data file.

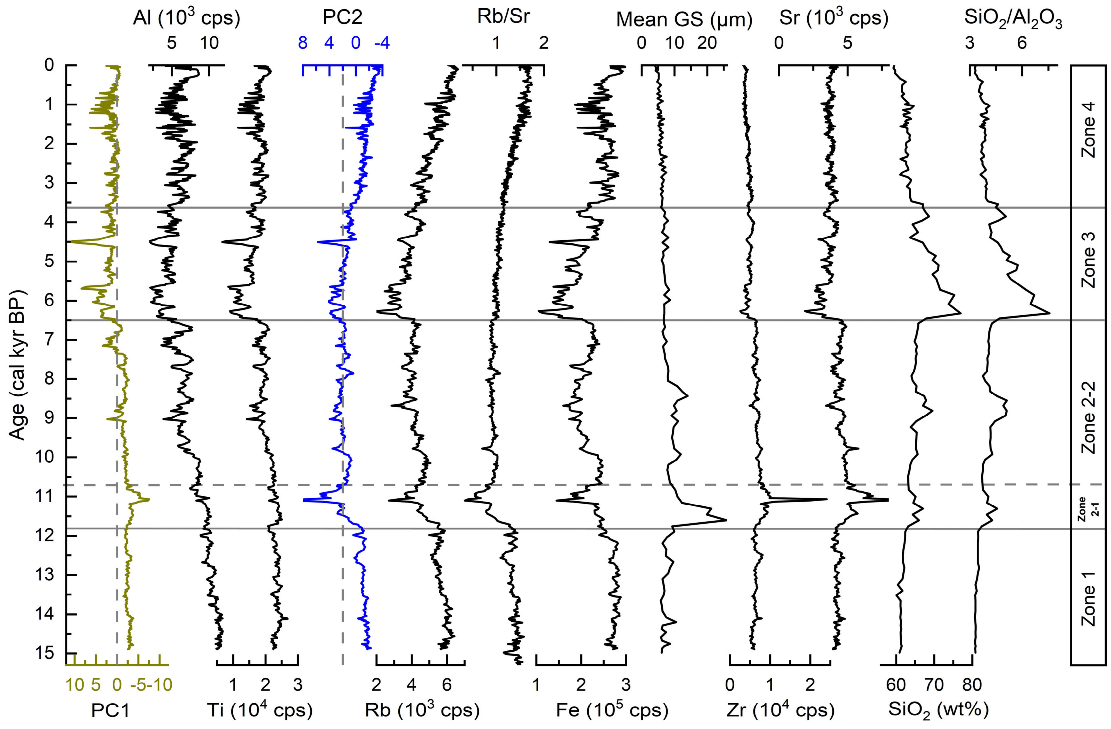

Supplementary Figure 3. Age profiles of selected geochemical elements determined by XRF-scanning and sample scores on PC1 (dark yellow curve) and PC2 (blue curve) of a principal component analysis (PCA) for core KNS15D from Kanas Lake. Geochemical zones determined by stratigraphically constrained incremental sum of squares (CONISS) are shown on the far right. Source data are provided as a Source Data file.

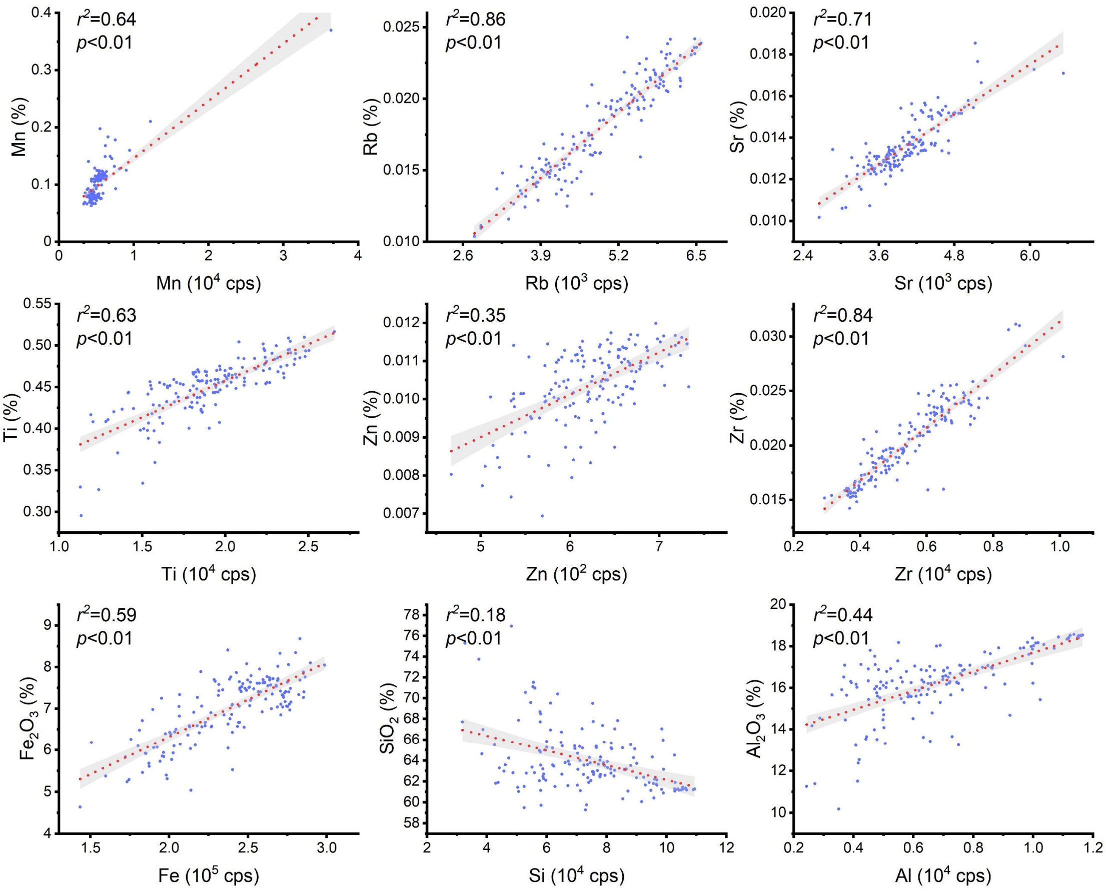

Supplementary Figure 4. Scatter plots and correlation results of selected XRF-scanning data and conventional XRF data (blue dots) from Kanas Lake. Significant variables were calculated using a two-tailed significance test at $p < 0.05$ based on Student's t-distribution, and the r^2 was adjusted accordingly. The red dashed line indicates the linear regression curve. The gray shaded bands are 95% confidence intervals. Source data are provided as a Source Data file.

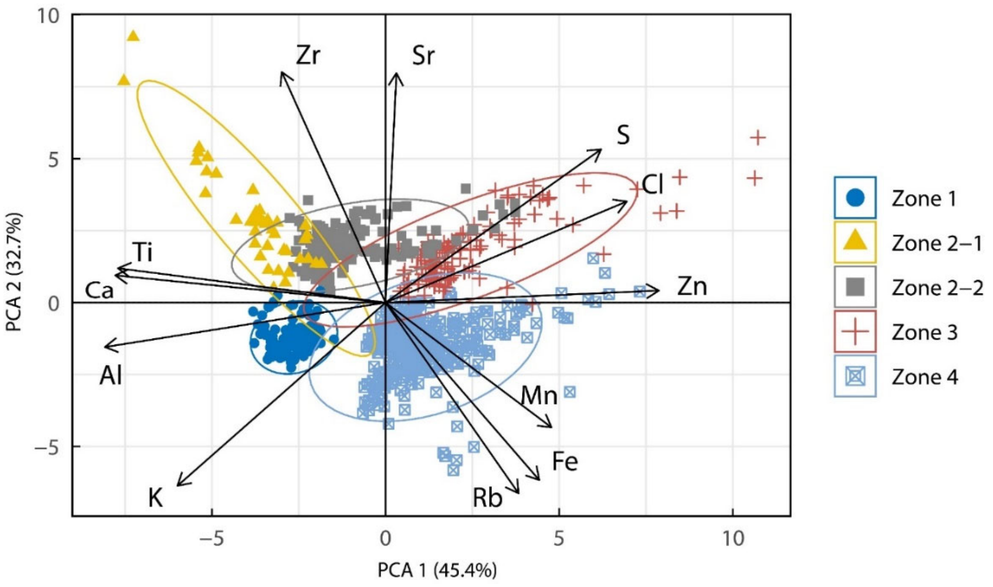

Supplementary Figure 5. Variable loadings and sample scores plotted on PC1 and PC2 of a principal component analysis (PCA) of selected XRF-scanning data from Kanas Lake. Source data are provided as a Source Data file.

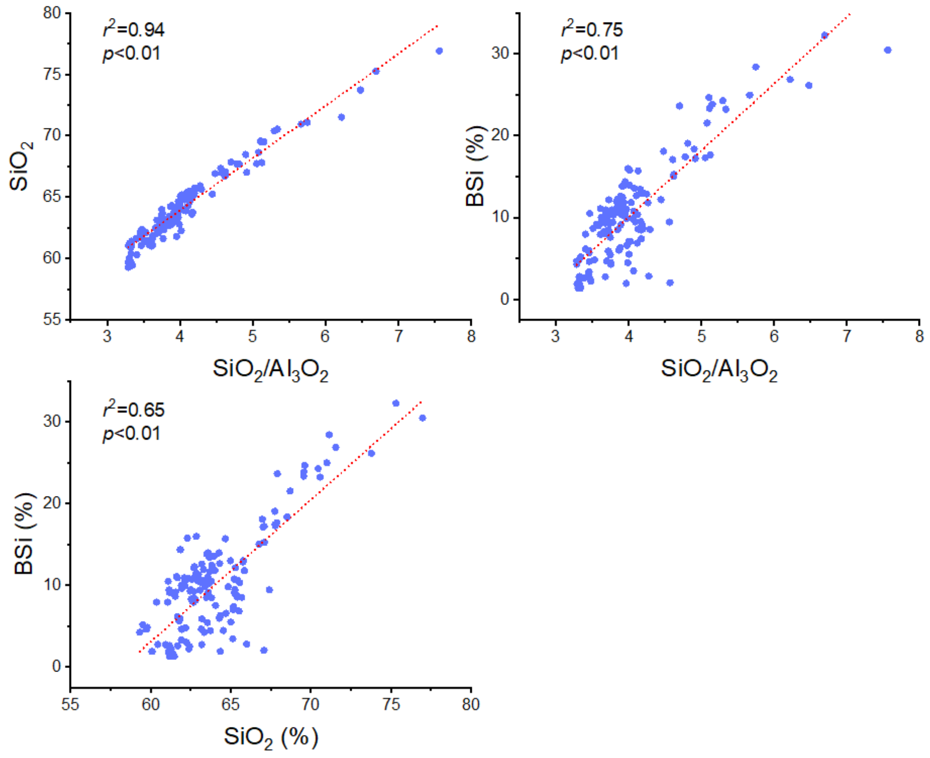

Supplementary Figure 6. Scatter plots and correlation results for the relationships between SiO_2/Al_3O_2, SiO_2 and biogenic silica (BSi) (blue dots). Significant variables were calculated using a two-tailed significance test at $p < 0.05$ based on Student's t-distribution, and the r^2 was adjusted accordingly. The red dashed line indicates the linear regression curve. Source data are provided as a Source Data file.

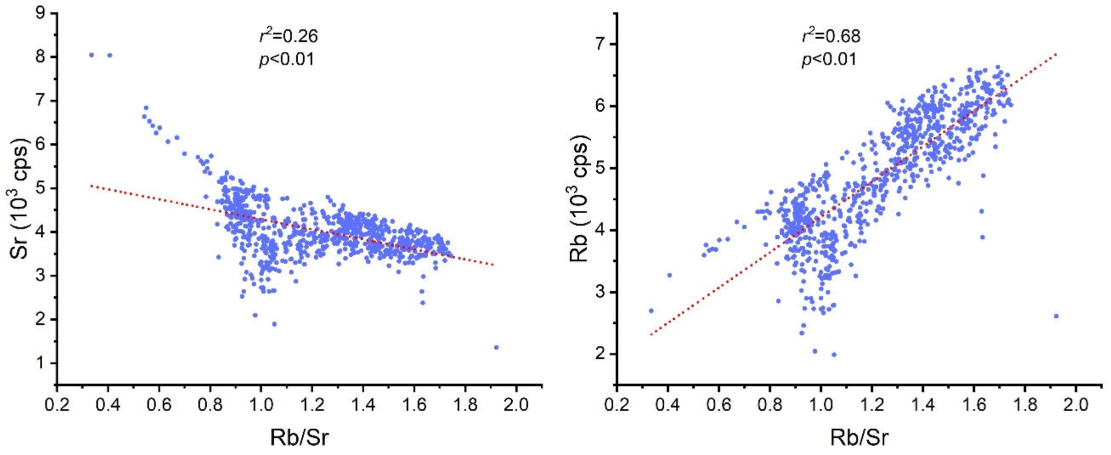

Supplementary Figure 7. Scatter plots and correlation results showing the relationship between Rb/Sr and Sr (left) and Rb (right) (blue dots) for Kanas Lake. Significant variables were calculated using a two-tailed significance test at p < 0.05 based on Student's t-distribution, and the r^2 was adjusted accordingly. The red dashed line indicates the linear regression curve. Source data are provided as a Source Data file.

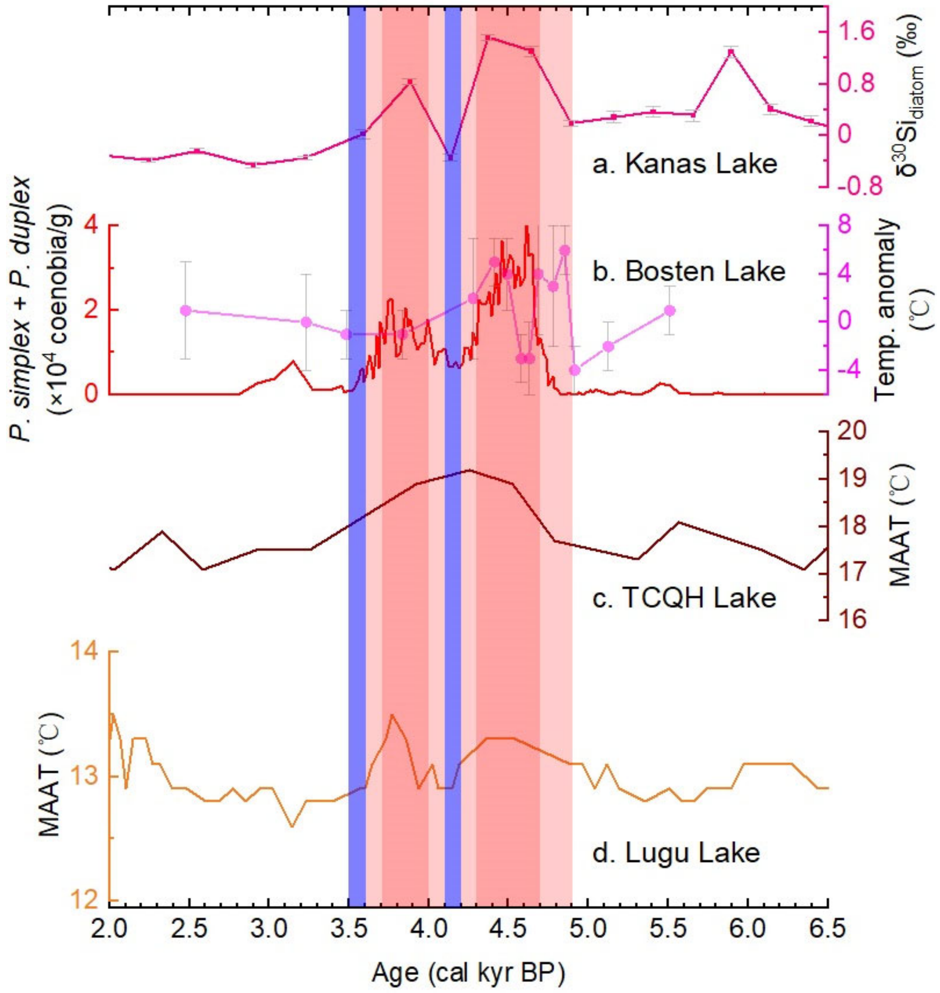

Supplementary Figure 8. Comparison of temperature reconstructions based on various proxies. (a) $\delta^{30}Si_{diatom}$ with 2-sigma absolute analytical errors from Kanas Lake during 6.5–2.0 kyr. (b) Sum of *P. simplex* + *P. duplex* coenobia and the temperature anomaly of clumped isotopes (Δ_{47}) with 1-sigma standard deviation from Bosten Lake, Xinjiang[38]. (c-d) Mean annual air temperature (MAAT) record from Lugu Lake and Tengchongqinghai (TCQH) Lake, on the southeastern margin of the TP[39]. The light red/blue shadings indicate warmer/colder intervals, while the red bars indicate the warmest stages. Source data are provided as a Source Data file.

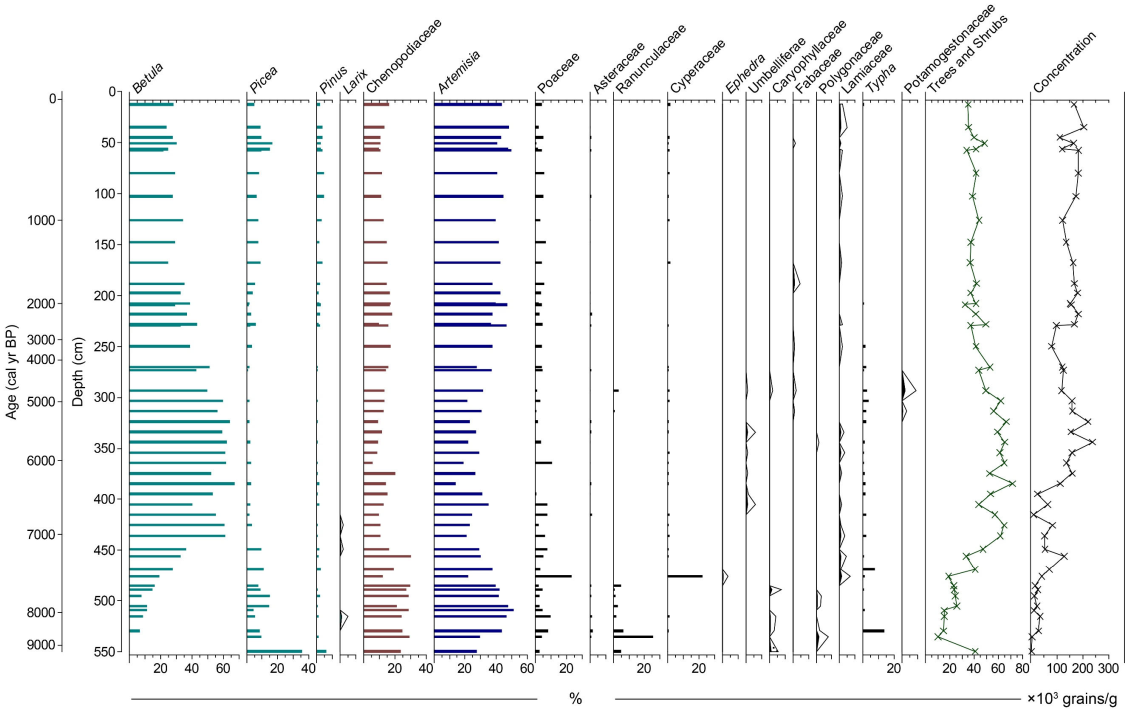

Supplementary Figure 9. Pollen diagram for core TWK15A from Tiewaike Lake (shading indicates 5 times exaggeration of scale). Source data are provided as a Source Data file.

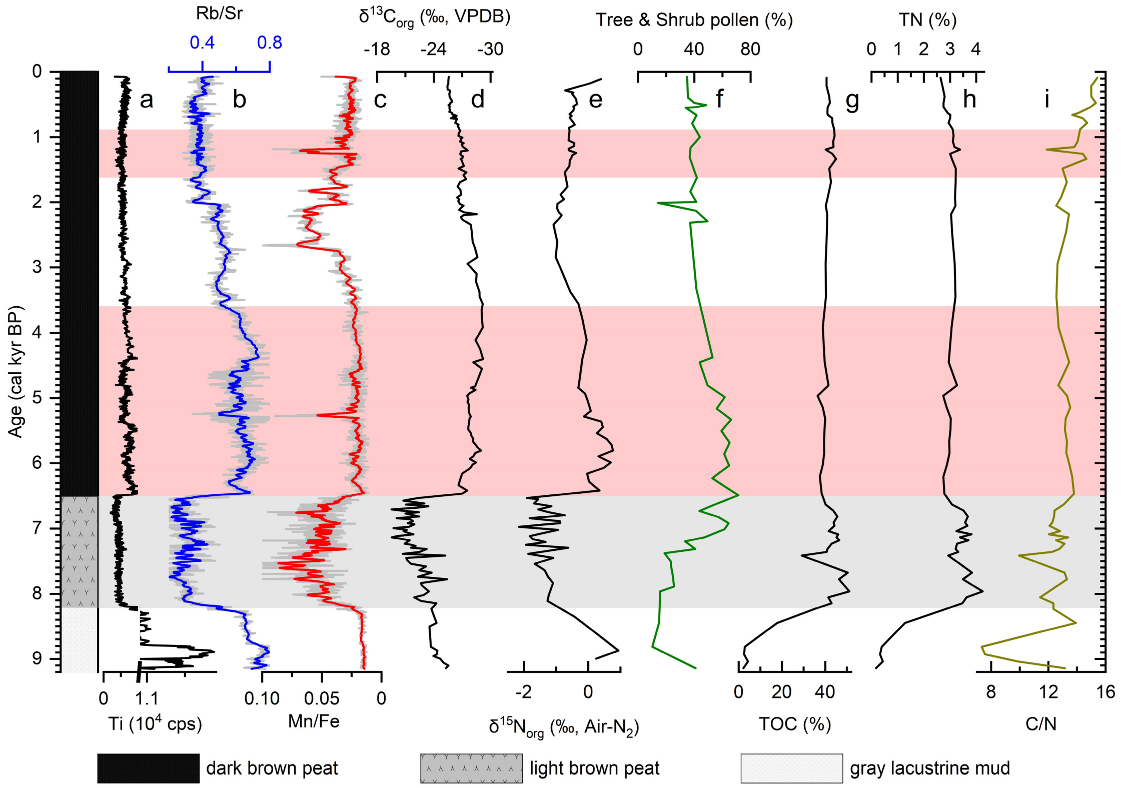

Supplementary Figure 10. Comparison of selected environmental proxies from core TWK15A from Tiewaike Lake. (a) Ti, (b) Rb/Sr (gray curve) with 21 points Savitzky-Golay filter smooth (blue curve), (c) Mn/Fe (gray curve) with 21 points Savitzky-Golay filter smooth (red curve), (d) $^{13}C_{org}$, (e) $\delta^{15}N_{org}$, (f) sum of tree and shrub pollen percentages (green curve), (g) total organic carbon (TOC), (h) total nitrogen (TN) and (i) C/N ratio (dark yellow curve). The red/gray shading indicates warmer/dryer intervals. Source data are provided as a Source Data file.

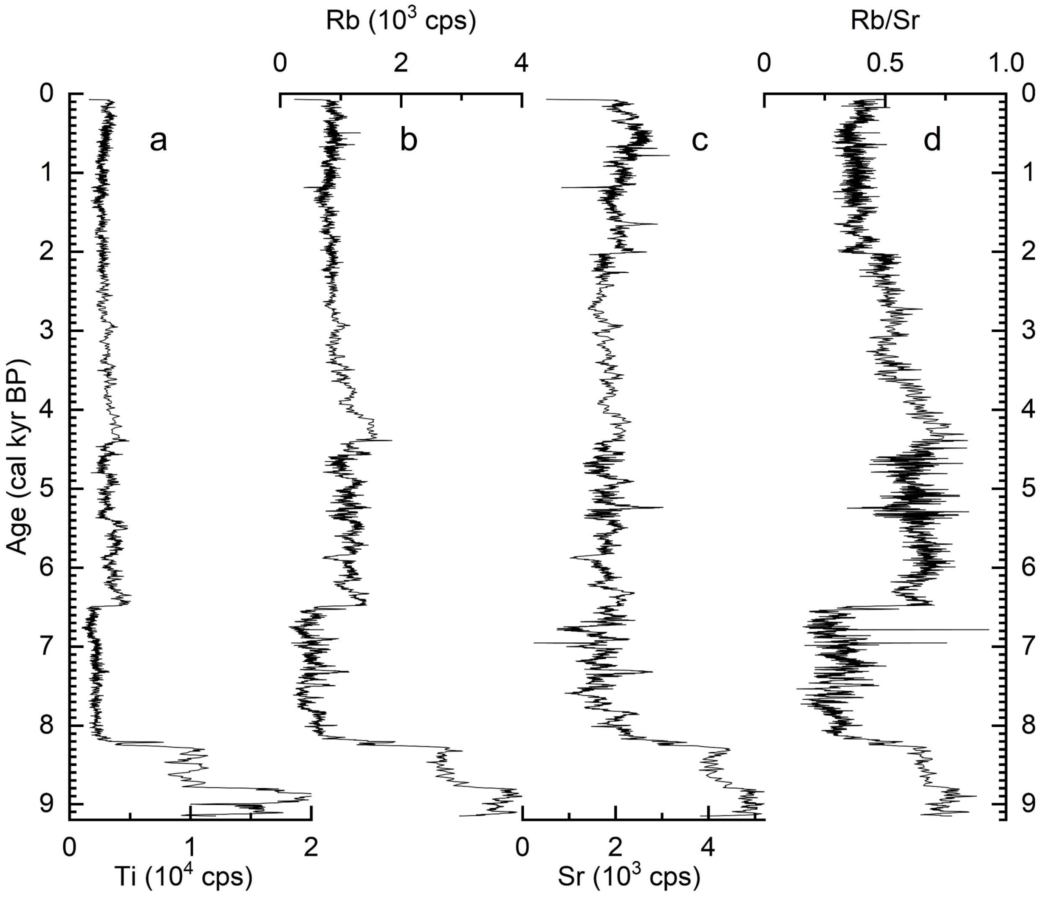

Supplementary Figure 11. Comparison of selected XRF elements from core TWK15A from Tiewaike Lake. (a)–(d) are Ti, Rb, Sr and Rb/Sr, respectively. Source data are provided as a Source Data file.

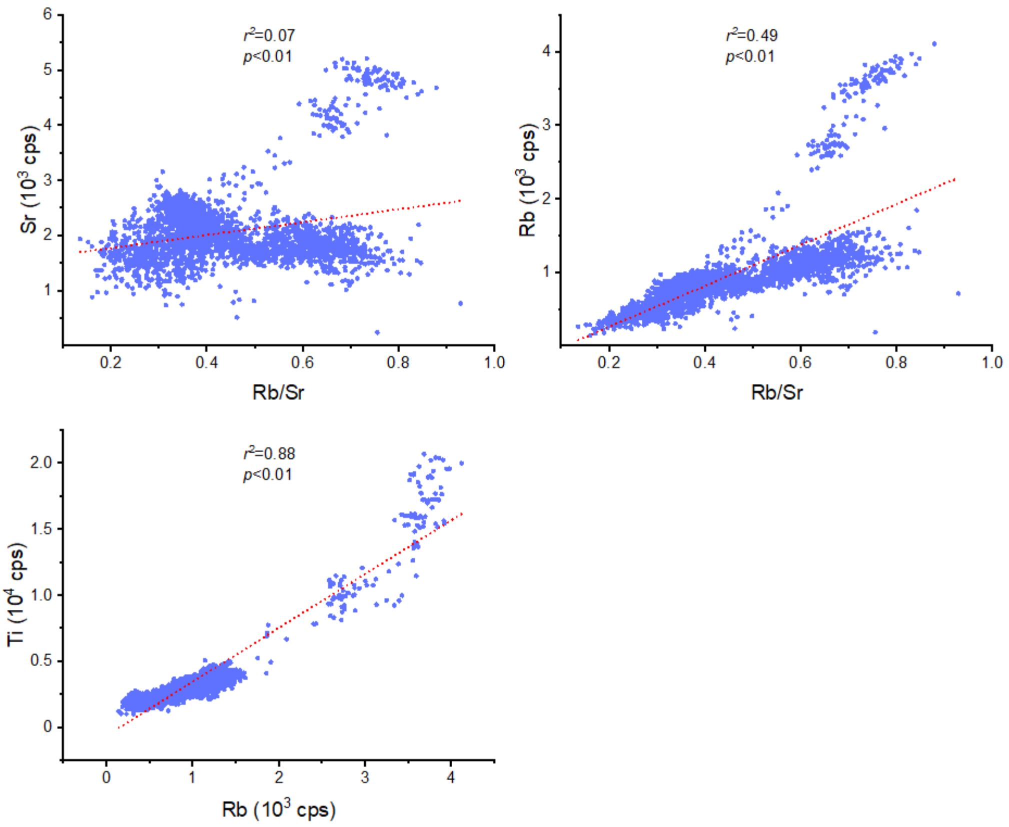

Supplementary Figure 12. Scatter plots and correlation results for the XRF-scanning data (Sr, Rb, Rb/Sr and Ti) (blue dots) from core TWK15A from Tiewaike Lake. Significant variables were calculated using a two-tailed significance test at $p < 0.05$ based on Student's t-distribution, and the r^2 was adjusted accordingly. The red dashed line indicates the linear regression curve. Source data are provided as a Source Data file.

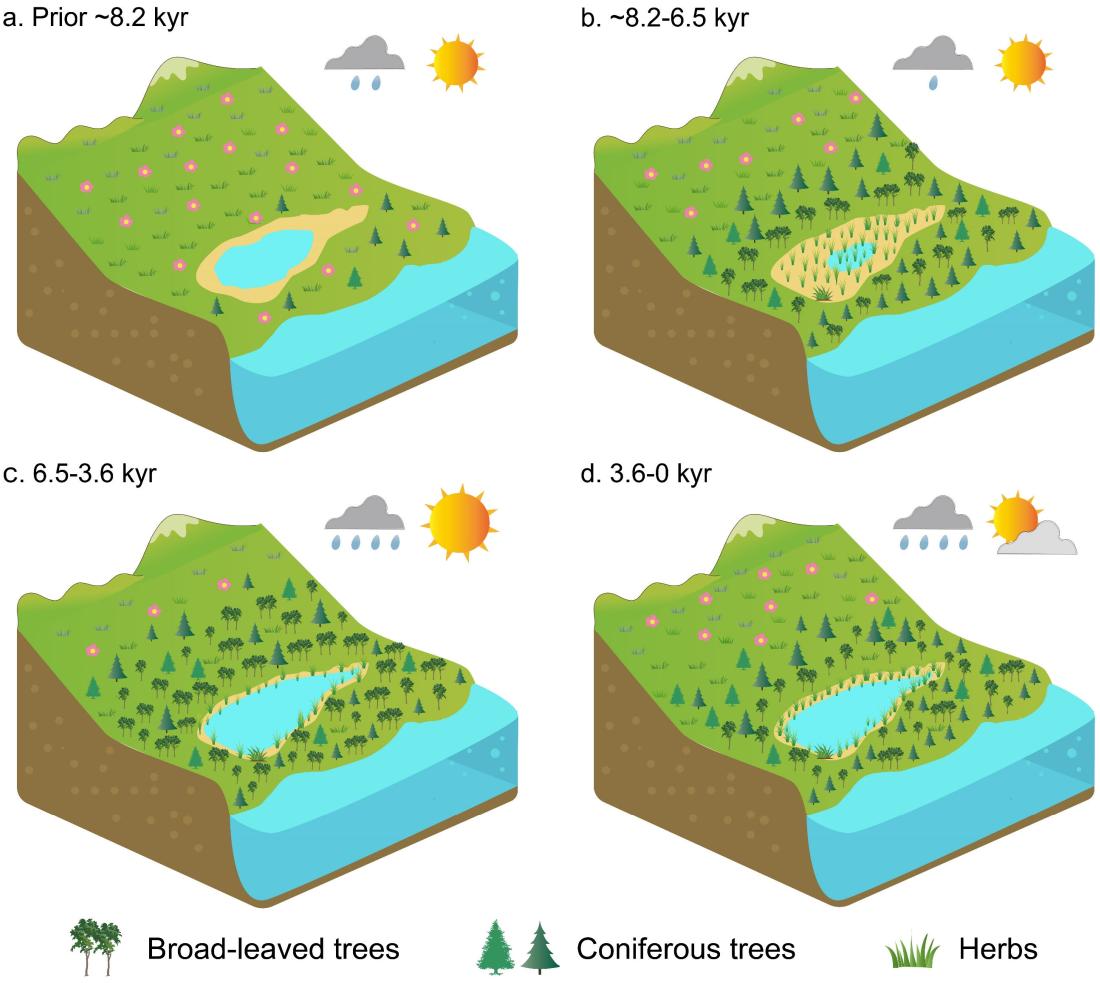

Supplementary Figure 13. Schematic model of stages in the paleoenvironmental evolution of Tiewaike Lake and its catchment (central blue polygon), and Kanas Lake and its catchment (right blue polygon). The four stages are: (a) prior to ~8.2 kyr, (b) ~8.2–6.5 kyr, (c) 6.5–3.6 kyr, and (d) 3.6–0 kyr. The graph was generated by L.X. using Adobe Illustrator 2021 on Microsoft Windows. Image credit: Kim Kraeer and Lucy Van Essen-Fishman, Integration and Application Network for the broad-leaved trees images (https://ian.umces.edu/media-library).

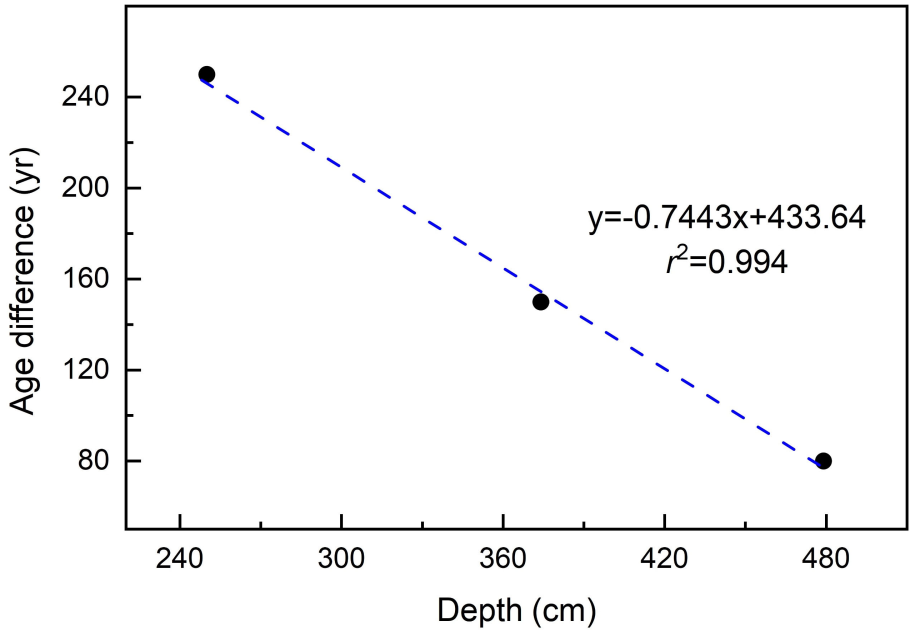

Supplementary Figure 14. Relationship between sediment depth and the age differences between the dating results for plant residues and bulk organic matter from the same stratigraphic horizons for core TWK15A from Tiewaike Lake. The blue dashed line indicates the linear regression curve. Note the strong linear relationship. Source data are provided as a Source Data file.

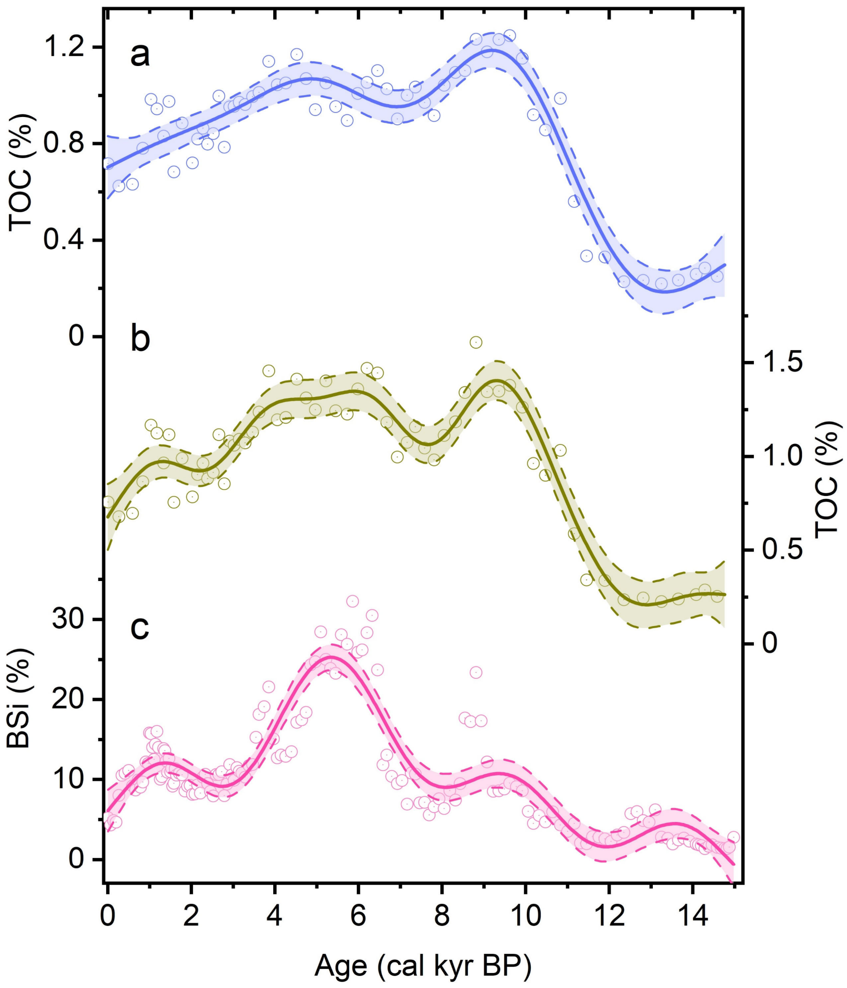

Supplementary Figure 15. Records of (a) uncorrected total organic carbon (TOC), and (b) TOC corrected by subtracting the BSi content (c) for sediment core KNS15D from Kanas Lake. All records are fitted with general additive models (GAMs). The shaded bands are 95% confidence intervals. Source data are provided as a Source Data file.

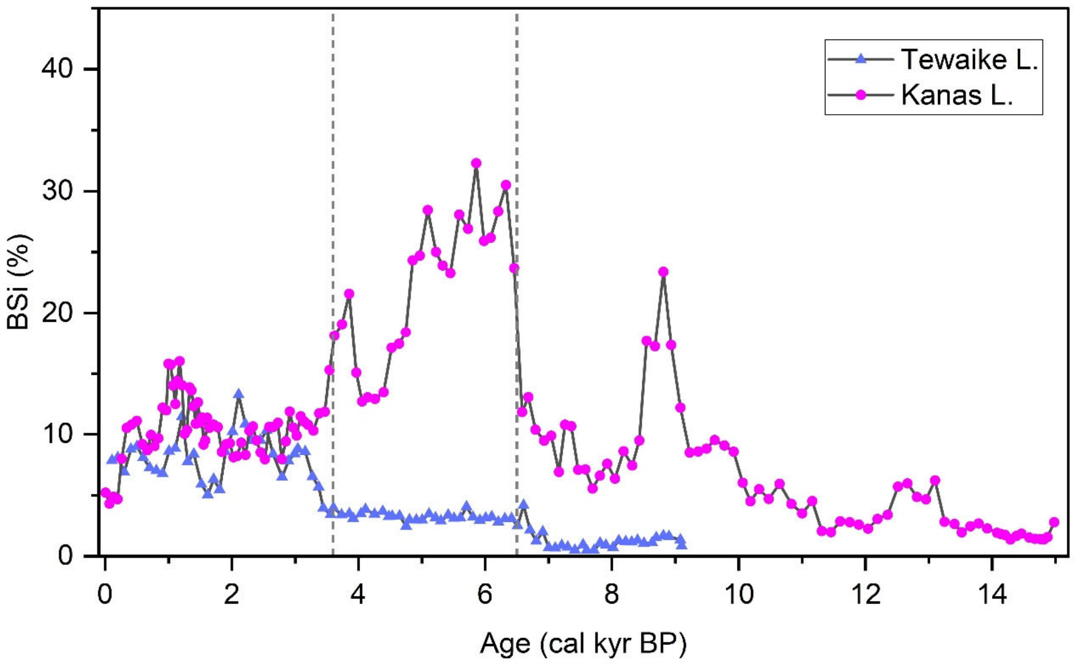

Supplementary Figure 16. Biogenic silica profile for core TWK15A from Tiewaike Lake and core KNS15D from Kanas Lake. Source data are provided as a Source Data file.

Supplementary Table 1. Radiocarbon dating results for core KNS15D from Kanas Lake.

Lab Code	Depth (cm)	Dating Material	$\delta^{13}C$ (‰)	^{14}C dates (yr BP)	Calibrated age (cal yr BP, 2σ, IntCal 20)
Beta411882	13.6	Tree twigs	-25.7	940±30	779-922
Beta411883	36	Plant material	-24.1	1780±30	1590-1738
Beta411884	48	Tree skin	-27.3	2390±30	2343-2674
Beta411885	62.5	Tree twigs	N/A	3160±30	3272-3451
Beta434432*	77	BOM	-24.8	4140±30	-
Beta434433*	89	BOM	-24.8	5990±30	-
Beta411887	106	Tree twigs	-31.6	7840±30	8542-8723
Beta434434*	117	BOM	-24.4	8750±30	-
Beta411888	146	Tree stem	-25.9	12340±40	14123-14821
Beta411889	151.5	Tree stem	-26.3	12220±40	14041-14310

Note: BOM: bulk organic matter. *Age was not used in this study.

Supplementary Table 2. Radiocarbon dating results for core TWK15A from Tiewaike Lake.

Lab Code	Depth (cm)	Dating Material	$\delta^{13}C$ (‰)	^{14}C dates (yr BP)	^{14}C dates-RE (yr BP)	Calibrated age (cal yr BP, 2σ, IntCal 20)
LZU18-163	39	BOM	-	1690±20	577±20	547-624
Beta434427	67	BOM	-25.6	1720±30	628±30	557-650
LZU18-164	96	BOM	-	1870±20	799±20	688-723
Beta427981	125	BOM	-26.5	2140±30	1090±30	956-1055
LZU18-165*	151	BOM	-	1950±20	920±20	-
LZU18-166	176	BOM	-	2470±20	1458±20	1310-1354
Beta434428	202	BOM	-26.8	2990±30	1997±30	1889-1988
LZU18-230	228	BOM	-	3210±20	2237±20	2160-2322
Beta428574	250	APR	-12.1	3810±30	3101±30	3255-3367
Beta427982	250	BOM	-29.0	4060±30	3101±30	3255-3367
LZU18-158	259	APR	-22.3	4120±20	3411±20	3591-3692
LZU18-159	272	APR	-21.7	4710±20	4001±20	4424-4515
Beta434429	342	BOM	-28.0	5790±30	4902±30	5591-5655
Beta428575	374	APR	-16.4	6160±30	5451±30	6210-6292
Beta427983	374	BOM	-23.4	6310±30	5451±30	6210-6292
LZU18-160	390	APR	-24.0	6580±20	5871±20	6665-6732
LZU18-161	475	APR	-22.2	7170±20	6461±20	7336-7385
Beta434918	479	APR	-21.1	7100±40	6391±40	7265-7417
Beta434430	479	BOM	-21.8	7180±30	6391±30	7269-7414
Beta427984	526	BOM	-23.4	8180±30	7429±30	8190-8320
Beta434431	538	BOM	-25.3	8860±30	8117±30	9002-9088

Note: BOM: bulk organic matter; APR: aquatic plant remains. *Age was rejected.

Supplementary References:

1. Wu, J., Liu, W., Zeng, H., Ma, L. & Bai, R. Water Quantity and Quality of Six Lakes in the Arid Xinjiang Region, NW China. *Environ. Process.* **1**, 115–125 (2014).
2. Xu, X., Yang, J., Dong, G., Wang, L. & Miller, L. OSL dating of glacier extent during the Last Glacial and the Kanas Lake basin formation in Kanas River valley, Altai Mountains, China. *Geomorphology* **112**, 306–317 (2009).
3. Li, Y. et al. Hydroclimatic changes over the past 900 years documented by the sediments of Tiewaike Lake, Altai Mountains, Northwestern China. *Quat. Int.* **452**, 91–101 (2017).
4. Huang, X. et al. Holocene Vegetation and Climate Dynamics in the Altai Mountains and Surrounding Areas. *Geophys. Res. Lett.* **45**, 6628–6636 (2018).
5. Cao, H., Huang X. & Xiang L. Soil erosion caused the increasing Holocene radiocarbon reservoir effect of Lake Kanas in the Altai Mountains. *Radiocarbon* **65**, 343–356 (2023).
6. de la Rocha, C.L., Brzezinski, M.A. & DeNiro, M.J. Fractionation of silicon isotopes by marine diatoms during biogenic silica formation. *Geochim. Cosmochim. Acta* **61**, 5051–5056 (1997).
7. Demarest, M.S., Brzezinski, M.A. & Beucher, C.P. Fractionation of silicon isotopes during biogenic silica dissolution. *Geochim. Cosmochim. Acta* **73**, 5572–5583 (2009).
8. Lin, X., Rioual, P., Peng, W., Yang, H. & Huang, X. Impact of recent climate change on Lake Kanas, Altai Mountains (N.W. China) inferred from diatom and geochemical evidence. *J. Paleolimnol.* **59**, 461–477 (2018).
9. Sun, M. Primary studies on the Holocene diatom assemblages and their paleoenvironmental significance in the Kanas Lake, Xinjiang (in Chinese with English abstract) (Lanzhou university, Lanzhou, 2020).
10. Cornelis, J.-T. et al. Tracing the origin of dissolved silicon transferred from various soil-plant systems towards rivers: a review. *Biogeosciences* **8**, 89–112 (2011).
11. Nantke, C.K.M., Frings, P.J., Stadmark, J., Czymzik, M. & Conley, D.J. Si cycling in transition zones: a study of Si isotopes and biogenic silica accumulation in the Chesapeake Bay through the Holocene. *Biogeochemistry* **146**, 145–170 (2019).
12. Panizzo, V.N., Swann, G.E.A., Mackay, A.W., Pashley, V. & Horstwood, M.S.A. Modelling silicon supply during the Last Interglacial (MIS 5e) at Lake Baikal. *Quat. Sci. Rev.* **190**, 114–122 (2018).
13. Swann, G.E.A. et al. Changing nutrient cycling in Lake Baikal, the world's oldest lake. *Proc. Natl. Acad. Sci.* **117**, 27211–27217 (2020).
14. Swann, G.E.A. et al. A combined oxygen and silicon diatom isotope record of Late Quaternary change in Lake El'gygytgyn, North East Siberia. *Quat. Sci. Rev.* **29**, 774–786 (2010).
15. Zahajská, P. et al. Impact of Holocene climate change on silicon cycling in Lake 850, Northern Sweden. *The Holocene* **31**, 1582–1592 (2021).
16. Chen, J. et al. Silicon isotope composition of diatoms as a paleoenvironmental proxy in Lake Huguangyan, South China. *J. Asian Earth Sci.* **45**, 268–274 (2012).
17. Davies, S.J., Lamb, H.F. & Roberts, S.J. Micro-XRF Core Scanning in Palaeolimnology: Recent Developments, in: Croudace, I.W., Rothwell, R.G. (Eds.), Micro-XRF Studies of Sediment Cores, Developments in Paleoenvironmental Research (Springer Netherlands, Dordrecht, 2015).
18. Stansell, N.D. et al. Abrupt Younger Dryas cooling in the northern tropics recorded in lake sediments from the Venezuelan Andes. *Earth Planet. Sci. Lett.* **293**, 154–163 (2010).
19. Olsen, J. et al. Lacustrine evidence of Holocene environmental change from three Faroese lakes: a multiproxy XRF and stable isotope study. *Quat. Sci. Rev.* **29**, 2764–2780 (2010).
20. Zeng, Y., Chen, J., Zhu, Z. & Li, J. Advance and Porspective of Rb / Sr Ratios in Lake Sediments as an Index of Paleoclimate / Paleoenvironment. *Adv. Earth Sci.* **26**, 805–810 (2011a).
21. Zeng, Y., Chen, J., Zhang, W., Zhu, Z. & Li, J. The non-residual Rb/Sr ratio of the Huguangyan Maar Lake and its implications for paleoclimate change. *Geochimica* **40**, 249–257 (2011b).
22. Kalugin, I. et al. 800-yr-long records of annual air temperature and precipitation over southern

Siberia inferred from Teletskoye Lake sediments. *Quat. Res.* **67**, 400–410 (2007).
23. Olsen, J., Anderson, N.J., Knudsen, M.F. Variability of the North Atlantic Oscillation over the past 5,200 years. *Nat. Geosci.* **5**, 808–812 (2012).
24. Davison, W. Iron and manganese in lakes. *Earth-Sci. Rev.* **34**, 119–163 (1993).
25. Haberzettl, T. et al. Lateglacial and Holocene wet—dry cycles in southern Patagonia: chronology, sedimentology and geochemistry of a lacustrine record from Laguna Potrok Aike, Argentina. *The Holocene* **17**, 297–310 (2007).
26. Yuan, K. et al. Responses of sedimentary proxy indicators to lake-level fluctuations on the central Tibetan Plateau since the last deglaciation. Prog. Phys. *Geogr. Earth Environ*. **46**, 922–948 (2022).
27. Meyers, P.A. & Teranes, J.L. Sediment Organic Matter, in: Last, W.M., Smol, J.P. (Eds.), Tracking Environmental Change Using Lake Sediments, Developments in Paleoenvironmental Research (Kluwer Academic Publishers, Dordrecht, 2002).
28. Smith, B.N. & Epsten, S. Two Categories of $^{13}C/^{12}C$ Ratios for Higher Plants. *Plant Physiol.* **47**, 380–384 (1971).
29. Wang, Y. et al. Temperature variations over the past 600 years documented by a δ13C record from terrestrial plant remains from Kanas Lake, Altai Mountains, Northwestern China. *Chin. Sci. Bull.* **62**, 2829–2839 (2017).
30. Oana, S. & Deevey, E.S. Carbon 13 in lake waters, and its possible bearing on paleolimnology. *Am. J. Sci.* **258**, 253–272 (1960).
31. Hong, B. et al. Increasing summer rainfall in arid eastern-Central Asia over the past 8500 years. *Sci. Rep.* **4**, 5279 (2015).
32. Feng, Z. et al. Vegetation changes and associated climatic changes in the southern Altai Mountains within China during the Holocene. *Holocene* **27**, 683–693 (2017).
33. Olsen, J., Anderson, N.J. & Leng, M.J. Limnological controls on stable isotope records of late-Holocene palaeoenvironment change in SW Greenland: a paired lake study. *Quat. Sci. Rev.* **66**, 85–95 (2013).
34. Wetzel, R.G. Limnology: Lake and River Systems (Academic Press, San Diego, 2001).
35. Talbot, M.R. & Johannessen, T. A high resolution palaeoclimatic record for the last 27 500 years in tropical West Africa from the carbon and nitrogen isotopic composition of lacustrine organic matter. *Earth Planet. Sci. Lett.* **110**, 23–37 (1992).
36. Gosling, W.D. et al. A stronger role for long-term moisture change than for CO_2 in determining tropical woody vegetation change. *Science* **376**, 653–656 (2022).
37. Reynolds, B.C. et al. An inter-laboratory comparison of Si isotope reference materials. *J. Anal. At. Spectrom.* **22**, 561–568 (2007).
38. Huang, X. et al. Sedimentary *Pediastrum* record of middle–late Holocene temperature change and its impacts on early human culture in the desert-oasis area of northwestern China. *Quat. Sci. Rev.* **265**, 107054 (2021).
39. Zhao, C. et al. Possible obliquity-forced warmth in southern Asia during the last glacial stage. *Sci. Bull.* **66**, 1136–1145 (2021).
