# Supplementary material for: Prehistoric population expansion in Central Asia promoted by the Altai Holocene Climatic Optimum
Source: Nat Commun. 2023 May 29;14:3102. doi: 10.1038/s41467-023-38828-4 (PMC10227073; doi:10.1038/s41467-023-38828-4)
Supplement: Supplementary file 3 — Description of Additional Supplementary Files [file 41467_2023_38828_MOESM3_ESM.pdf]

Description of Additional Supplementary Files:

Supplementary Data 1: This dataset contains Summed probability distribution (SPD) of published original radiocarbon dates from archaeological sites before 0 CE in this study. The dataset includes Dates (n=2955) and Bins (n=1983) for the study area as well as the Altai Mountains and the surrounding areas with Dates (n=411) and Bins (n=292). The dataset also includes location information and references for the archaeological sites collected by this study.
