# Supplementary material for: Prehistoric population expansion in Central Asia promoted by the Altai Holocene Climatic Optimum
Source: Nat Commun. 2023 May 29;14:3102. doi: 10.1038/s41467-023-38828-4 (PMC10227073; doi:10.1038/s41467-023-38828-4)
Supplement: Supplementary file 5 — Reporting Summary [file 41467_2023_38828_MOESM5_ESM.pdf]

nature portfolio

Corresponding author(s): Xiaozhong Huang and Virginia N. Panizzo

Last updated by author(s): May 5, 2023

Reporting Summary

Nature Portfolio wishes to improve the reproducibility of the work that we publish. This form provides structure for consistency and transparency in reporting. For further information on Nature Portfolio policies, see our Editorial Policies and the Editorial Policy Checklist.

Statistics

For all statistical analyses, confirm that the following items are present in the figure legend, table legend, main text, or Methods section.

n/a	Confirmed	
☐	☒	The exact sample size (*n*) for each experimental group/condition, given as a discrete number and unit of measurement
☐	☒	A statement on whether measurements were taken from distinct samples or whether the same sample was measured repeatedly
☐	☒	The statistical test(s) used AND whether they are one- or two-sided<br>*Only common tests should be described solely by name; describe more complex techniques in the Methods section.*
☒	☐	A description of all covariates tested
☒	☐	A description of any assumptions or corrections, such as tests of normality and adjustment for multiple comparisons
☐	☒	A full description of the statistical parameters including central tendency (e.g. means) or other basic estimates (e.g. regression coefficient) AND variation (e.g. standard deviation) or associated estimates of uncertainty (e.g. confidence intervals)
☐	☒	For null hypothesis testing, the test statistic (e.g. *F*, *t*, *r*) with confidence intervals, effect sizes, degrees of freedom and *P* value noted<br>*Give P values as exact values whenever suitable.*
☒	☐	For Bayesian analysis, information on the choice of priors and Markov chain Monte Carlo settings
☒	☐	For hierarchical and complex designs, identification of the appropriate level for tests and full reporting of outcomes
☒	☐	Estimates of effect sizes (e.g. Cohen's *d*, Pearson's *r*), indicating how they were calculated

Our web collection on statistics for biologists contains articles on many of the points above.

Software and code

Policy information about availability of computer code

Data collection	Canadian Archaeological Radiocarbon Database (CARD 2.0) (https://www.canadianarchaeology.ca);<br>Google earth (https://www.earth.google.com/);<br>WorldClim 2.0 (https://www.worldclim.org/);<br>China Meteorological data service center (http://www.data.cma.cn/)
Data analysis	R software (v4.0.5) and RStudio software (Version 1.2.5001). The source code of Bacon and GAM model are obtained from Blaauw & Christen (2011) and Simpson (2018), respectively, which described in Method section. The source code of PCA are obtained from Factoshiny 2.4 packages at website https://mirrors.sjtug.sjtu.edu.cn/cran/web/packages/Factoshiny/index.html.<br>Microsoft Excel (Microsoft);<br>Tilia 2.11 software;<br>ArcGIS 10.2;<br>Origin 2022b;<br>OxCal v.4.4.4.

For manuscripts utilizing custom algorithms or software that are central to the research but not yet described in published literature, software must be made available to editors and reviewers. We strongly encourage code deposition in a community repository (e.g. GitHub). See the Nature Portfolio guidelines for submitting code & software for further information.

Data

Policy information about availability of data

All manuscripts must include a data availability statement. This statement should provide the following information, where applicable:

- Accession codes, unique identifiers, or web links for publicly available datasets
- A description of any restrictions on data availability
- For clinical datasets or third party data, please ensure that the statement adheres to our policy

Source data are provided with this paper. The base-maps used in Fig. 1 are accessible through the Geospatial Data Cloud website (https://www.gscloud.cn/) and Google Earth website (https://www.earth.google.com/). The base-maps used in Fig. 3 and Fig. 4 are accessible through the Natural Earth website (https://www.naturalearthdata.com/downloads/10m-raster-data/). Pollen samples used in this study are stored at Key Laboratory of Western China's Environmental Systems (Ministry of Education), Lanzhou University and can be obtained from the corresponding authors (xzhuang@lzu.edu.cn) upon reasonable request.

Human research participants

Policy information about studies involving human research participants and Sex and Gender in Research.

Reporting on sex and gender	n/a
Population characteristics	n/a
Recruitment	n/a
Ethics oversight	n/a

Note that full information on the approval of the study protocol must also be provided in the manuscript.

Field-specific reporting

Please select the one below that is the best fit for your research. If you are not sure, read the appropriate sections before making your selection.

☐ Life sciences ☐ Behavioural & social sciences ☒ Ecological, evolutionary & environmental sciences

For a reference copy of the document with all sections, see nature.com/documents/nr-reporting-summary-flat.pdf

Ecological, evolutionary & environmental sciences study design

All studies must disclose on these points even when the disclosure is negative.

Study description	We combined new chronological, palaeoecological and geochemical evidence, including 31 C-14 dates, high stratigraphic resolution scanning X-ray fluorescence measurements, and analyses of grain size, stable isotopes of organic carbon and nitrogen, total organic carbon, total nitrogen, biogenic silica, and silicon isotopes of diatom silica, to provide a detailed characterization of climate change in the middle and late Holocene, for a region that is under-represented in the literature. Combined with archaeological evidence (2955 C-14 dates from 1118 archaeological sites), we construct a narrative of the climatic forcing of human population migration and cultural exchange in Central Asia.
Research sample	The research samples included 156 sub-samples in core KNS15D and 504 sub-samples in core TWK15A. The chronology from two cores are spanning 15 kyr and 9.1 kyr for core KNS15D and core TWK15A, respectively.
Sampling strategy	We sampled the sediments of Kanas Lake and Tiewaike Lake using a UWITEC piston corer. 31 plant macrofossil/bulk organic matter samples were conducted by radiocarbon dating with accelerator mass spectrometry. 46 diatom silicon isotope samples were analyzed in wet plasma mode using the high mass-resolution capability of a Thermo Scientific Neptune Plus MC-ICP-MS (multi-collector inductively coupled plasma mass spectrometer). 156 BSi samples were determined using the molybdate blue method. A total of 49 samples were used for pollen analysis following standard HCl–NaOH–HF treatment. Samples for analyses of total organic carbon (TOC), total nitrogen (TN) and stable isotope ratios (δ13Corg, δ15Norg) were pre-treated with 1 N HCl at 60 °C to remove carbonate, washed with deionised water, and freeze-dried prior to analysis, and then these samples were measured using an online Conflo III-Delta Plus isotope ratio mass spectrometer combined with a Flash EA1112 elemental analyzer. Element compositions of the sediments were determined by core scanning X-ray fluorescence (XRF-scanning), at a 2-mm resolution, using an Avaatech XRF core scanner.
Data collection	Huang X. and Xiang L. conducted the fieldwork. Sun M. conducted the silicon isotope analyses at the British Geological Survey, UK and recorded the results using Microsoft Excel 2016 on Microsoft Windows. Xiang L. and Huang X. conducted part of the laboratory work and recorded it using Microsoft Excel 2016 on Microsoft Windows. Xiang L., Zheng M., and Huang C., collected the archaeological radiocarbon dates and downloaded via Microsoft Edge v. 112.0.1722.64. All data were stored in Microsoft Excel files on Microsoft Windows.

Timing and spatial scale	We sampled the core sediments of Kanas Lake (KNS15D, 48°43′19.55″N, 87°1′3.98″E) and Tiewaike Lake (TWK15A, 48°49′36.58N, 87°00′55.54″E) during January to March of 2015. All data are collected between 2015-2021 for lab analysis. The spatial scale of sampling was based on the site scale, with sediment samples collected from multiple depths in each lake. Additionally, we collected archaeological radiocarbon dates from northern central Asia, which provide important chronological context for our study of environmental changes in the region.
Data exclusions	Three samples of bulk organic matter (BOM) in core KNS15D were excluded from the age model due to the carbon reservoir effect in lacustrine sediments. The exclusion criteria is whether the dated sample contains carbon reservoir effect.
Reproducibility	For diatom silicon isotope samples, a minimum of two analytical replicates were made per sample, with repeated sampling of the standard (diatomite) to validate the data and sample bracketing with standard NBS28 to correct for any instrumental drift. All attempts at replication were successful.
Randomization	n/a
Blinding	We did not use different treatment for our samples, therefore blingding was not used.

Did the study involve field work? ☒ Yes ☐ No

Field work, collection and transport

Field conditions	Field work was conducted in the winter because it was easier to drill cores when the lakes were covered with ice. Theoretically, our field is not restricted by climatic conditions and could be done at any time of the year.
Location	The sedimentary cores of Kanas Lake (KNS15D, 48°43′19.55″N, 87°1′3.98″E) and Tiewaike Lake (TWK15A, 48°49′36.58N, 87°00′55.54′′E) are located in southern Altai Mountains, Xinjiang Province, China. We obtained verbal permission from Kanas Lake National Nature Reserve, Xinjiang Province, China. Therefore, it does not require any official issuing authority and permission.
Access & import/export	Our samples were collected with permission, following local and national laws. We obtained oral permission from Kanas Lake National Nature Reserve.
Disturbance	Our filed sampling will not disturb the natural landscape and environment. The core sediments were not disturbed during the field work and lab work.

Reporting for specific materials, systems and methods

We require information from authors about some types of materials, experimental systems and methods used in many studies. Here, indicate whether each material, system or method listed is relevant to your study. If you are not sure if a list item applies to your research, read the appropriate section before selecting a response.

Materials & experimental systems

n/a	Involved in the study	
☒	☐	Antibodies
☒	☐	Eukaryotic cell lines
☐	☒	Palaeontology and archaeology
☒	☐	Animals and other organisms
☒	☐	Clinical data
☒	☐	Dual use research of concern

Methods

n/a	Involved in the study	
☒	☐	ChIP-seq
☒	☐	Flow cytometry
☒	☐	MRI-based neuroimaging

Palaeontology and Archaeology

Specimen provenance	Samples and data were collected independently by our team, following local and national laws. We obtained oral permission from Kanas Lake National Nature Reserve, Xinjiang Province, China. Therefore, it does not require any official issuing authority and permission.
Specimen deposition	The pollen samples were stored in the Key Laboratory of Western China's Environmental Systems (Ministry of Education), College of Earth and Environmental Sciences, Lanzhou University, and are available to researchers for relevant research purposes unpon request.
Dating methods	31 plant macrofossil/bulk organic matter samples were conducted by radiocarbon dating. Accelerator mass spectrometry (AMS) C-14 dates from 7 samples of terrestrial plant macrofossils and 3 samples of bulk organic matter (BOM) were obtained from core

KNS15D; the analyses were conducted by Beta Analytic Inc. (Florida, USA). Due to the carbon reservoir effect in lacustrine sediments, 3 samples of BOM were excluded from the age model. For Tiewaike Lake, 21 dates (14 from BOM and 7 from aquatic plant macrofossils) were obtained from core TWK15A; the analyses were conducted by Beta Analytic Inc. and Lanzhou University. The AMS 14C dating results were calibrated to calendar years before present (BP, before 1950 CE) using the Bacon package (Blaauw and Christen, 2011) with the IntCal 20 calibration dataset (Reimer et al., 2020).

☒ Tick this box to confirm that the raw and calibrated dates are available in the paper or in Supplementary Information.

Ethics oversight

No ethical approval was required for non-conflict-interest sedimentary samples collected by our funded research.

Note that full information on the approval of the study protocol must also be provided in the manuscript.
